# Effects of elevated temperature, reduced hydroperiod, and invasive bullfrog larvae on pacific chorus frog larvae

**DOI:** 10.1371/journal.pone.0265345

**Published:** 2022-03-15

**Authors:** Bailey R. Tasker, Karli N. Honebein, Allie M. Erickson, Julia E. Misslin, Paul Hurst, Sarah Cooney, Skylar Riley, Scott A. Griffith, Betsy A. Bancroft

**Affiliations:** 1 Department of Biology, Gonzaga University, Spokane, Washington, United States of America; 2 Department of Environmental Studies and Sciences, Gonzaga University, Spokane, Washington, United States of America; 3 Department of Mathematics and Computer Science, Whitworth University, Spokane, Washington, United States of America; Universitat Zurich, SWITZERLAND

## Abstract

Climate change and invasive species threaten many ecosystems, including surface freshwater systems. Increasing temperatures and reduced hydroperiod due to climate change may promote the persistence of invasive species and facilitate new invasions due to potentially higher tolerance to environmental stress in successful invaders. Amphibians demonstrate high levels of plasticity in life history characteristics, particularly those species which inhabit both ephemeral and permanent water bodies. We tested the influence of two projected effects of climate change (increased temperature and reduced hydroperiod) on Pacific chorus frog (*Pseudacris regilla*) tadpoles alone and in combination with the presence of tadpoles of a wide-spread invasive amphibian, the American bullfrog (*Lithobates catesbeianus*). Specifically, we explored the effects of projected climate change and invasion on survival, growth, mass at stage 42, and development rate of Pacific chorus frogs. Direct and indirect interactions between the invasive tadpole and the native tadpole were controlled via a cage treatment and were included to account for differences in presence of the bullfrog compared to competition for food resources and other direct effects. Overall, bullfrogs had larger negative effects on Pacific chorus frogs than climate conditions. Under future climate conditions, Pacific chorus frogs developed faster and emerged heavier. Pacific chorus frog tadpoles developing in the presence of American bullfrogs, regardless of cage treatment, emerged lighter. When future climate conditions and presence of invasive American bullfrog tadpoles were combined, tadpoles grew less. However, no interaction was detected between climate conditions and bullfrog presence for mass, suggesting that tadpoles allocated energy towards mass rather than length under the combined stress treatment. The maintenance of overall body condition (smaller but heavier metamorphs) when future climate conditions overlap with bullfrog presence suggests that Pacific chorus frogs may be partially compensating for the negative effects of bullfrogs via increased allocation of energy towards mass. Strong plasticity, as demonstrated by Pacific chorus frog larvae in our study, may allow species to match the demands of new environments, including under future climate change.

## Introduction

Human impacts have dramatically altered many ecosystems, including surface freshwater habitats [1]. Freshwater systems are influenced by climate change in multiple ways, including increased rate of surface evaporation, reduced hydroperiods, alteration of chemical and biological processes, and increased temperatures [2]. Additionally, invasive species pose a threat to these same habitats. Invasive species can demonstrate high levels of phenotypic plasticity and adaptation potential, allowing them to respond to environmental change and successfully invade new ranges [3]. Moreover, invasive species may have higher tolerance to environmental stress than native species [4, 5]. This plasticity and ability to tolerate changes in environmental conditions suggest that invasive species may flourish under future climate conditions. Therefore, climate change and invasive species can interact to negatively affect native species.

In freshwater systems, amphibians represent a taxonomic group that may be especially sensitive to climate change and invasion. Climate change is expected to have wide ranging effects on amphibian species, including range shifts, changes in life history characteristics, and changes in interactions between species [6, 7]. These effects can vary across species [8], and even within a species the effects can vary by ontogenetic stage and population [9]. Similarly, invasive species can have large negative effects on amphibian survival, reproduction, development, and distribution [10–15]. As with climate change, the effects of an invasion depend on specifics of the habitat, as well as the identity of both the native and invasive species [12, 15].

However, amphibians are highly plastic in morphology and life history and can respond to stressors in complex ways [16]. Certain combinations of stressors can produce synergistic effects on tadpoles, such that the effect of two combined stressors is larger than the sum of each effect alone [17–20]. Behavioral responses can include increased or decreased activity in the presence of predators or competitors [21, 22] and altered microhabitat use [23]. In the larval stage, morphological responses can include changes in tail fin depth and length that may have important consequences for swimming speed and performance [24]. Faster progress towards metamorphosis has been observed in response to several environmental conditions including high risk predator treatments [25], increased competition [26], altered hydroperiod [27], and increased temperatures [28]. However, rapid development may have negative consequences [16, 29]. Overall, many species demonstrate some level of developmental plasticity, which is hypothesized to reduce the negative effects of climate change on amphibians.

Pacific chorus frogs (*Psuedacris regilla*) have a broad distribution in western North America, ranging from Baja California, Mexico through British Columbia, Canada and are often considered generalist species. Pacific chorus frogs currently inhabit and breed in a wide range of habitat types from large, permanent ponds to small puddles and exhibit phenotypic plasticity in response to environmental cues [30], including increased temperatures and reduced hydroperiods. For example, Pacific chorus frogs developed more quickly under warmer conditions, but emerged at a smaller size (both in snout-vent length (SVL) and mass [8]. Similarly, drying conditions due to reduced hydroperiod resulted in increased development rate for Pacific chorus frogs [31].

American bullfrogs (*Lithobates catesbeianus*; hereafter “bullfrogs”), native to the Southeastern United States, are invasive in many countries around the world. Bullfrog invasion has been implicated in the decline of other amphibian species [32] and they are considered one of the most problematic of amphibian invaders worldwide [15]. American bullfrogs typically breed in permanent ponds in both their native and invasive ranges [33]. Adult bullfrogs can act as predators on several life history stages of other amphibian species, including those of Pacific chorus frogs [34, 35]. Bullfrog larvae can act as predators or competitors with other amphibian larvae, often reducing the survival and growth of native amphibian larvae [36–38]. The effects of larval bullfrogs on survival in larval Pacific chorus frogs are unclear, with at least one study finding reduced chorus frog survival in the presence of overwintered larval bullfrogs [39] while other studies do not detect reduced survival [40, 41]. Several studies observed a negative effect of bullfrog larvae on chorus frog development rate [12, 42] and growth [40, 43]. The degree and nature of the interaction between bullfrogs and Pacific chorus frogs may drive the differences in effects observed in different experiments. For example, Kupferberg [37] found negative effects of larval bullfrogs on Pacific chorus frog tadpole survival in small containers, but did not observe negative effects of bullfrogs on chorus frog tadpoles in more realistic field enclosures. Thus, allowing chorus frog tadpoles to avoid direct interaction with bullfrogs may reduce the negative effects of bullfrogs on chorus frog tadpoles.

In the US Pacific Northwest, bullfrogs are limited to low elevation sites, likely in part by temperature [44]. These two species are documented living in the same ponds in several low-elevation locations [12, 45]. Climate change may shift the suitable habitat for bullfrogs into higher elevation sites, resulting in increased co-occurrence of these two species. In addition, large scale shifts in environmental conditions associated with climate change are known to affect the outcome of species interactions. Competitive relationships may become more or less intense as breeding phenology or growth rates shift in response to climate change [46], suggesting that future interactions between these two species may change with altered environmental conditions.

Here, we tested the individual and combined effects of simulated future climate conditions (warmer water, reduced hydroperiod) and species invasion (overwintered bullfrog tadpoles) on survival, growth, and development of Pacific chorus frogs. We further tested the effects of direct contact between Pacific chorus frogs and bullfrogs using a cage treatment for the invasive species. We hypothesized that simulated future climate conditions (warmer water, reduced hydroperiod) would accelerate the rate of development in Pacific chorus frogs but reduce size (mass and length) at metamorphosis. Further, we hypothesized that the presence of bullfrog larvae would reduce survival, growth, and progress towards metamorphosis, but only in the treatment allowing direct contact (open cage). When both stressors (future climate and invasion) were combined, we hypothesized a synergistic interaction between the two stressors, specifically when bullfrogs were allowed to directly interact with chorus frogs.

## Methods

### Animal collection

Pacific chorus frog larvae were collected from a temporary pond in the Dishman Hills Natural Area of Spokane County, WA, USA. This population was assumed to be naïve to bullfrogs, as no known populations of bullfrogs are near this site (>5 km to nearest permanent waterbody) and the pond is a temporary pond which does not support breeding populations of bullfrogs due to their extended larval period at higher latitudes within their range [44]. Bullfrog larvae were collected from Fish Lake near Cheney, WA, USA. After field collection, Pacific chorus frog and bullfrog larvae were kept in groups of five or six, separately by species, in 9.5-L holding tanks with water conditioned with NovAqua Plus and Amquel Plus (Kordon) and fed *ad libitum* daily with a 3∶1 mixture (by volume) of rabbit chow to fish flakes. Tanks were kept in a climate-controlled room set to 14°C and a 14∶10 photoperiod. Five days prior to the start of the experiment, tank temperatures were cycled from 14°C at night to 19°C during the day to re-acclimate tadpoles to diurnal temperature shifts. Animals were collected under appropriate permits SCP Bancroft 18–141 and an Aquatic Invasive Species permit from Washington State Department of Fish and Wildlife.

### Experimental design

**Fig 1:**
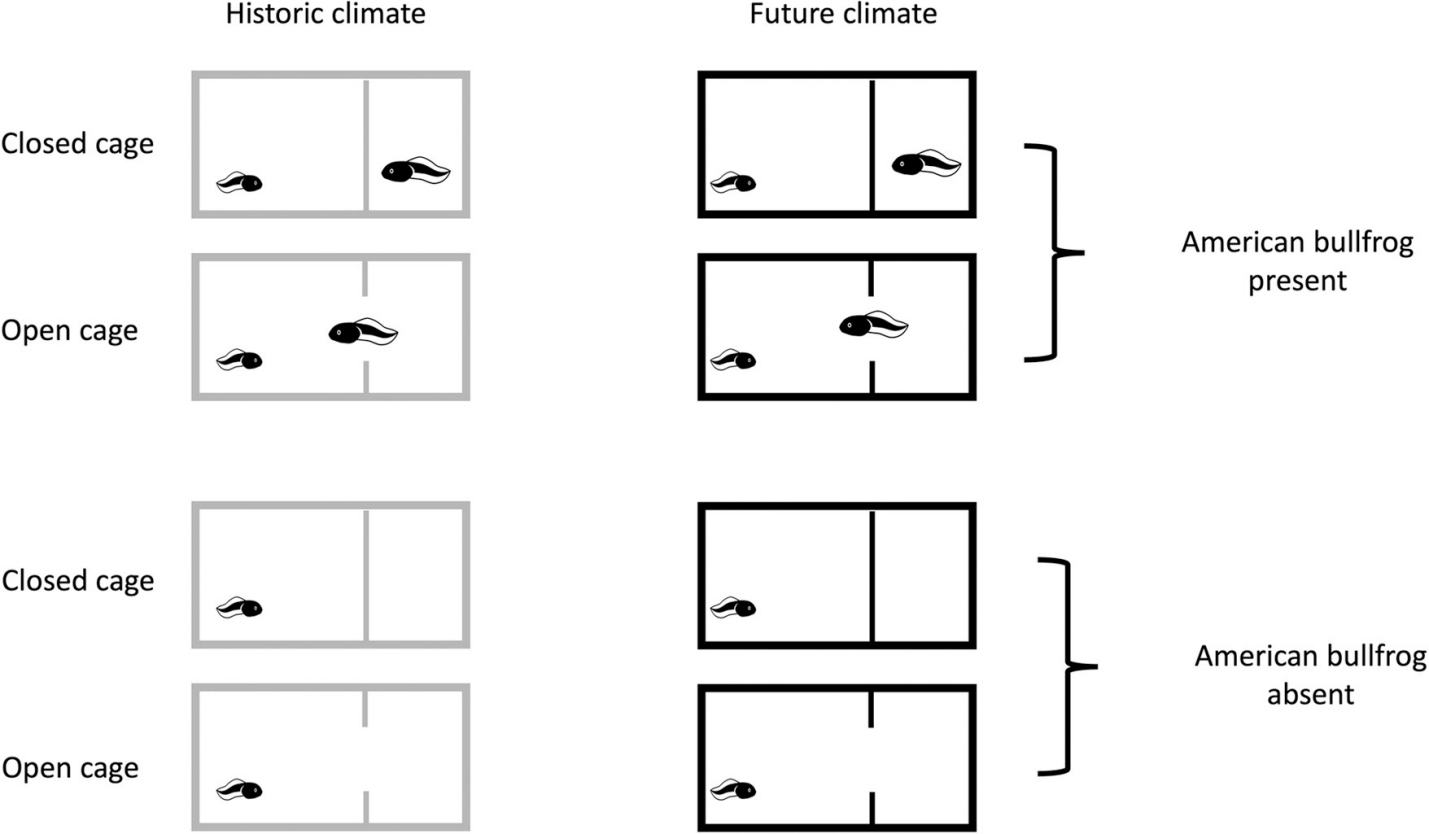
Representation of eight treatment groups in the experimental design. The smaller tadpole represents a single Pacific chorus frog (*Pseudacris regilla*) larva, while the larger tadpole represents a single American bullfrog (*Lithobates catesbeianus*) larva. Dividing lines within the tanks represent the cage treatment (open or closed). Tadpole images are from artist Parkjisun, sourced from The Noun Project website, under a Creative Commons-BY license.

The experiment was a 2 × 2 × 2 factorial design which consisted of a climate treatment with two levels (current climate and future climate), and an invasive species treatment with two levels (absence of invasive species and presence of invasive species), and an interaction treatment with two levels (caged and uncaged; Fig 1). In total, we had eight treatment groups with six replicates of each treatment (N = 48). Glass tanks (9.5L; 31 cm x 16 cm x 20 cm) were randomly assigned to treatment groups and were placed on wire racks in a temperature-controlled room set to 12°C with a 14:10 photoperiod. Each tank was filled to 15 cm deep with tap water treated with NovAqua Plus and AmQuel Plus (Kordon). A single Pacific chorus frog tadpole was randomly assigned and added to each tank after being photographed for subsequent image analysis. Although chorus frog tadpoles are often found in groups in the field, using one chorus frog tadpole per tank allowed us to precisely monitor survival, growth, and development of individual tadpoles and alleviated the need for an additional treatment group (density).

Throughout the experiment, animals were fed 3:1 rabbit chow to fish flakes daily (*ad libitum*). Waste was removed as needed from tanks, along with partial water changes as needed to maintain water quality. Animals were checked daily and dead tadpoles removed and photographed. This study was carried out in strict accordance with requirements of Gonzaga University’s Institutional Animal Care and Use Committee (IACUC), according to standards set by the U.S. National Science Foundation, The U.S. National Institutes of Health, The U.S. Department of Agriculture, and Gonzaga University. All work was approved in 2018 by Gonzaga University’s Animal Care and Use Committee, which does not issue permit numbers. All personnel were trained in animal care procedures. At the beginning of the study, a humane endpoint was determined such that any animals which were listless (not responsive to disturbance in the tank) and not feeding would be removed from the experiment and euthanized immediately using buffered MS-222. No animals were observed to qualify for this endpoint, though a total of six tadpoles died during the experiment. No direct cause of death could be determined for any tadpole. Dead bullfrog tadpoles (a total of two during the experiment) were replaced with a new bullfrog, but dead Pacific chorus frogs were not replaced.

#### Climate treatments

**Fig 2:**
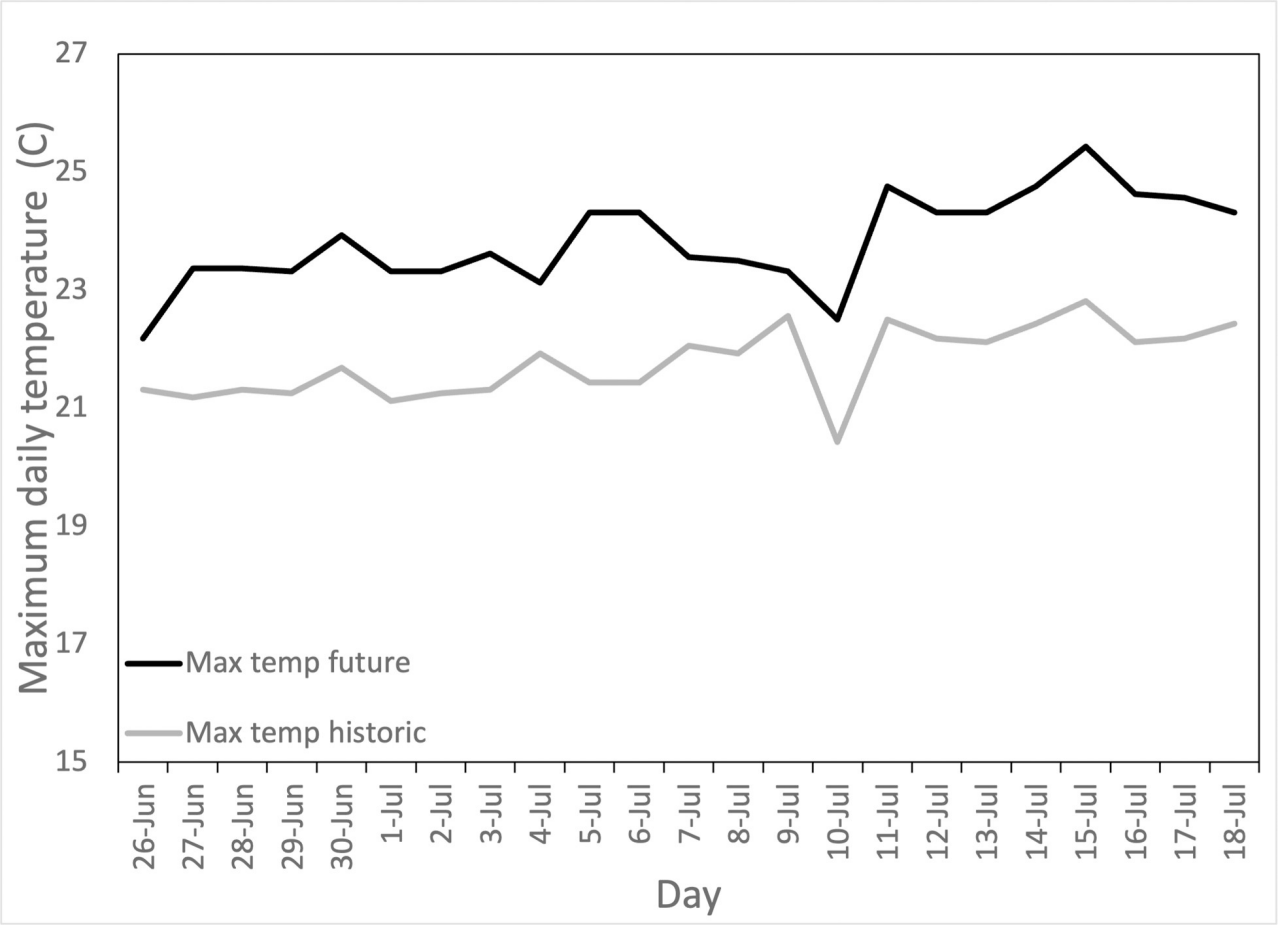
Representative daily measured maximum temperature in a historic treatment tank and a future treatment tank. Daily maximum measured temperatures did not always reach the daily set temperature due to power issues with heaters (see [42]), but a consistent difference between the treatment groups was maintained. Temperature scenarios were designed to provide variation in daily maximum temperature, while maintaining an overall positive slope to represent the increase in average daily temperature over the summer months.

To simulate climate change in temporary ponds, we manipulated both water temperature and hydroperiod simultaneously. We generated two distinct climate scenarios to represent historic conditions and potential future conditions (S1 Appendix). In the future climate treatments, the daily maximum temperature was 2.24°C warmer and the daily minimum temperature was 1.45°C warmer than historic climate treatments, with day-to-day variation to simulate natural conditions. (Fig 2). The daily minimum temperature was reached at 8 am each day, with the tank steadily warming to reach the daily maximum temperature at 6 pm. This pattern reflects the diurnal temperature cycles observed in local ponds. A distributed network of microprocessors (Arduino Nano) and small computers (Raspberry Pi) recorded and controlled the temperature of each tank. Each tank’s temperature was monitored by a single waterproof temperature probe (Maxim DS18B20) and heated by a power-modulated 50W aquarium heater (ViaAqua) connected to the distributed system [47].

Every week we removed water from all tanks to simulate evaporation loss, withdrawing more water in the future climate scenarios [48], such that by week four, the historic tanks were 10.2 cm deep while the future were 7.1 cm deep (S1 Appendix). Water that was lost through evaporation was replaced as needed to maintain the weekly level for each treatment.

#### Invasive species and cage treatments

To test the effects of the invasive bullfrogs on chorus frogs, a single overwintered bullfrog tadpole was introduced to half of the tanks. As we were interested in the difference between physical (direct) and visual/chemical (indirect) interactions between bullfrogs and chorus frogs, we also included an interaction treatment using a cage. All tanks had a piece of standard fiberglass window screen (21 cm x 17 cm) affixed to the bottom and sides of the tank with silicone caulk 10 cm from one end, separating 1/3 of the tank from the rest of the tank. In half of the tanks (open cage treatment), a window (13 cm x 6.5 cm) was cut in the screen and placed so the bottom of the opening was flush with the bottom of the tank. In the absence of bullfrog tadpoles, the closed tanks served as controls for the presence of the screen and the smaller swimming area available. Food was available on both sides of the mesh divider in all bullfrog treatment groups.

### Data collection and analysis

Pacific chorus frogs were removed from the tanks at Gosner stage 42 (forelimb emergence; [49]), photographed, weighed, and euthanized with an overdose of buffered MS-222 (tricaine methanesulfonate; Sigma). We measured start length and end length (at stage 42) for each tadpole using ImageJ [50]. We measured the body length (snout-vent length, SVL) for both the start and end length measurements. To measure growth, we calculated both absolute relative growth and relative growth rate. Absolute relative growth was calculated as the proportion of the initial body size (SVL) that was added during the experimental period ((final SVL -intial SVL)/intial SVL) [51]. We then calculated the daily relative growth rate by dividing absolute relative growth by the number of days each tadpole was in the experimental conditions (days until stage 42). To track progress through metamorphosis, we recorded the number of experimental days until stage 42. All Pacific chorus frogs either metamorphosed or died by day 34, at which point the experiment was terminated.

We used generalized linear models (GLMs) to test the effects of our treatments on Pacific chorus frog survival, mass, and days to stage 42. We used logistic regression (binomial GLM with logit link) to assess patterns in survival. As mass data cannot be negative values, we used a Gaussian GLM with a log link. Development (days to stage 42) was modeled using a quasi-Poisson GLM with a log link. We did not stage tadpoles prior to the start of the experiment to avoid handling stress, so we used the starting length as a covariate in the mass and days to stage 42 models to account for differences in starting size and developmental stage at the beginning of the experiment. Absolute relative growth and was modeled using analysis of covariance (ANCOVA), while relative growth rate was modeled using analysis of variance (ANOVA) via the aov function in R. To account for the varying number of days each tadpole was exposed to experimental conditions, we included days to metamorphosis as a covariate in the absolute relative growth model. Fully factorial models were used, including all two-and three-way experimental factor (climate, invasion, cage) interactions. Furthermore, interactions with starting length in the mass and development models and days in the absolute relative growth model were included in initial models. If the interaction terms with the covariates (starting length or days) were non-significant (indicating a lack of evidence for heterogeneous slopes among groups), these interactions were not included in the final model, though the covariate was retained as a main effect, along with all main effects and two- and -three way interactions among experimental factors. R statistical software (version 4.1.1) was used for all analyses and graphing [52]. For all GLM models, analysis of deviance based on likelihood ratio Chi-square tests was used to assess the significance of terms using the Anova function in the car package for R [53]. Likelihood ratios (rather than Wald tests) were used due to small sample sizes [54]. Model assumptions were assessed using residual plots. When appropriate, post hoc analyses were conducted using the emmeans package [55]. Alpha levels were set to 0.05 for all comparisons. All graphs were created using packages ggplot2 [56] and patchwork [57].

## Results

Four Pacific chorus frog tadpoles died before reaching stage 42 (two in future climate, bullfrog present, closed cage treatment; one in future climate, bullfrog present, open cage treatment; one in historic climate, bullfrog absent, closed cage treatment). Due to the scarcity of events, we used Firth-adjusted estimates [58] implemented in the brglm2 package [59] and did not include interactions in our model (e.g., only main effects were included). No effects of any treatment on survival were detected (Table 1A, regression results in Table A in S2 File). All tadpoles that died before reaching stage 42 were excluded from further analyses.

**Table 1:** Analysis of deviance table for all generalized linear regression models of treatment effects on survival, mass, and development.

Model	Source	Likelihood ratio	df	p (Chi-square)
A) Survival	Climate	-0.4498	1	1.000
	Invasive	1.30527	1	0.2533
	Cage	-0.47783	1	1.000
	Start body length	-0.59362	1	1.000
B) Mass	**Climate**	**4.1068**	**1**	**0.04371**
	**Invasive**	**4.6617**	**1**	**0.030843**
	**Cage**	**6.9667**	**1**	**0.008304**
	**Start body length**	**10.2634**	**1**	**0.001357**
	Climate*Invasive	0.0701	1	0.791209
	Climate*Cage	0.7222	1	0.395411
	Invasive*Cage	0.7496	1	0.386614
	Climate*Invasive*Cage	0.1255	1	0.723167
C) Development	**Climate**	**22.4549**	**1**	**0.000002**
	Invasive	0.1055	1	0.7454
	Cage	0.9600	1	0.3272
	**Start body length**	**16.4055**	**1**	**0.000051**
	Climate*Invasive	0.8824	1	0.3475
	Climate*Cage	0.3731	1	0.5413
	Invasive*Cage	0.3621	1	0.5473
	Climate*Invasive*Cage	0.0507	1	0.8218

**Fig 3:**
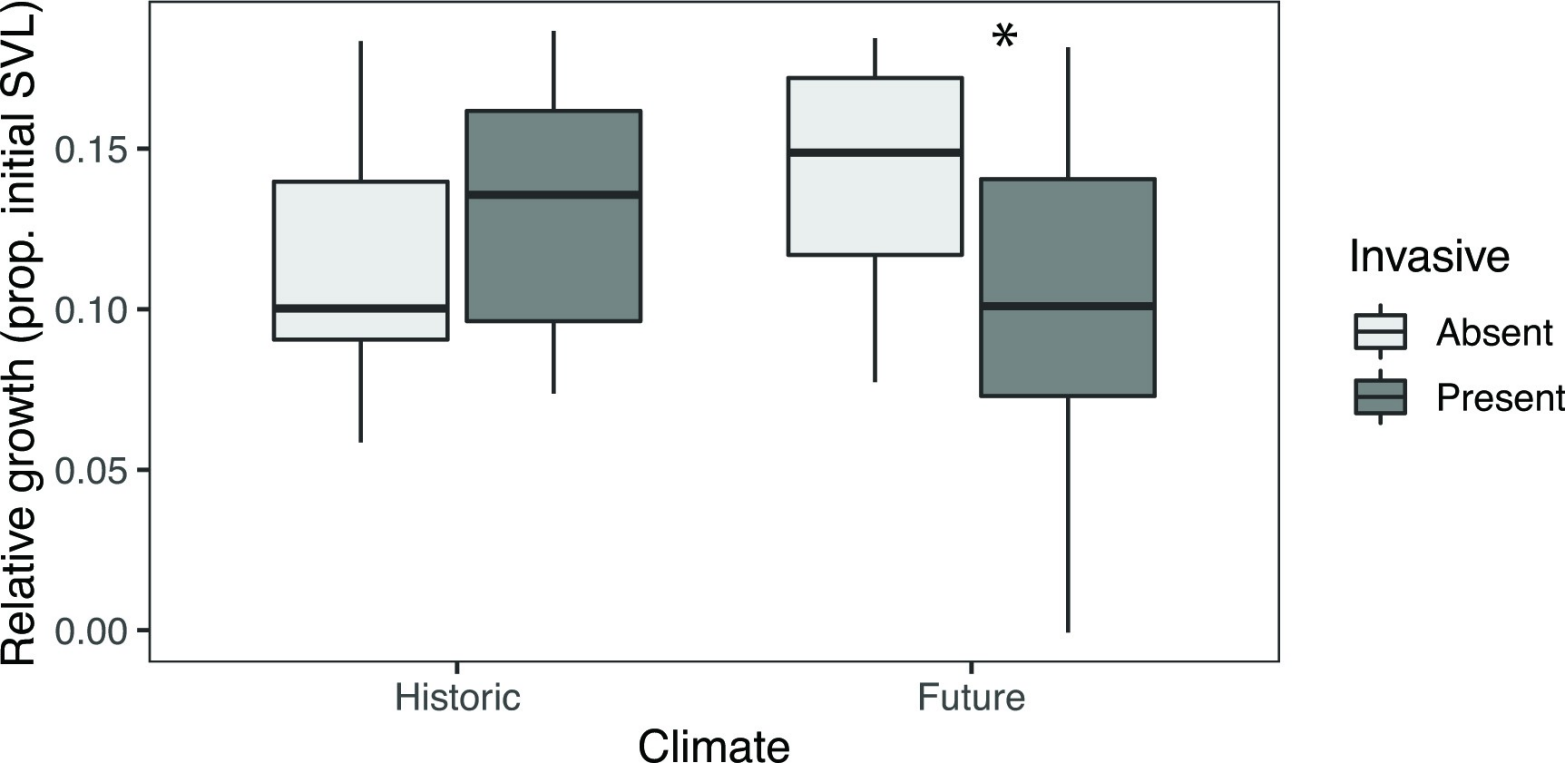
Bullfrog larvae reduced the absolute relative growth of Pacific chorus frog larvae under simulated future conditions. Relative growth represents the proportion of the initial body length (snout-vent length [SVL]) obtained during the experimental period. Under historic (cooler, less evaporation) conditions, no effect of bullfrogs was observed on growth of chorus frogs. Under future conditions, bullfrog presence reduced growth of chorus frog larvae (t-ratio = 2.254; df = 2,35; p = 0.0304; marked with an asterisk in plot). Boxplots are standard boxplots, with median and standard quartiles ± 1.5*IQR whiskers.

Absolute growth, but not growth rate, was affected by climate and invasion. Evidence for an interaction between climate treatment and invasive species presence was detected for absolute growth (Table 2). In future climates, chorus frog absolute growth was higher when bullfrogs were absent (t-ratio = 2.254, df = 2,35, p = 0.0304; Fig 3). Chorus frog tadpole relative growth rate was not affected by any treatment. However, marginal evidence for an interaction between climate treatment and invasive species presence was detected (F-value = 4.090; df = 1,36; p = 0.0506), such that the same pattern was observed in future climates for absolute growth rate as for absolute growth.

**Table 2:** Analysis of covariance for absolute relative growth (proportion of initial length) of Pacific chorus frogs.

Source	df	SS	MS	F	p (Chi-square)
Climate	1	0.0	0.01	0.001	0.9732
Invasive	1	9.8	9.84	0.854	0.3617
Cage	1	3.6	3.56	0.309	0.5819
Days	1	5.5	5.54	0.481	0.4924
Climate*Invasive	**1**	**56.4**	**56.40**	**4.896**	**0.0335**
Climate*Cage	1	1.0	0.96	0.084	0.7742
Invasive*Cage	1	20.4	20.38	1.769	0.1921
Climate*Invasive*Cage	1	14.8	14.85	1.289	0.2640
Residuals	36	403.1	11.52		

**Fig 4:**
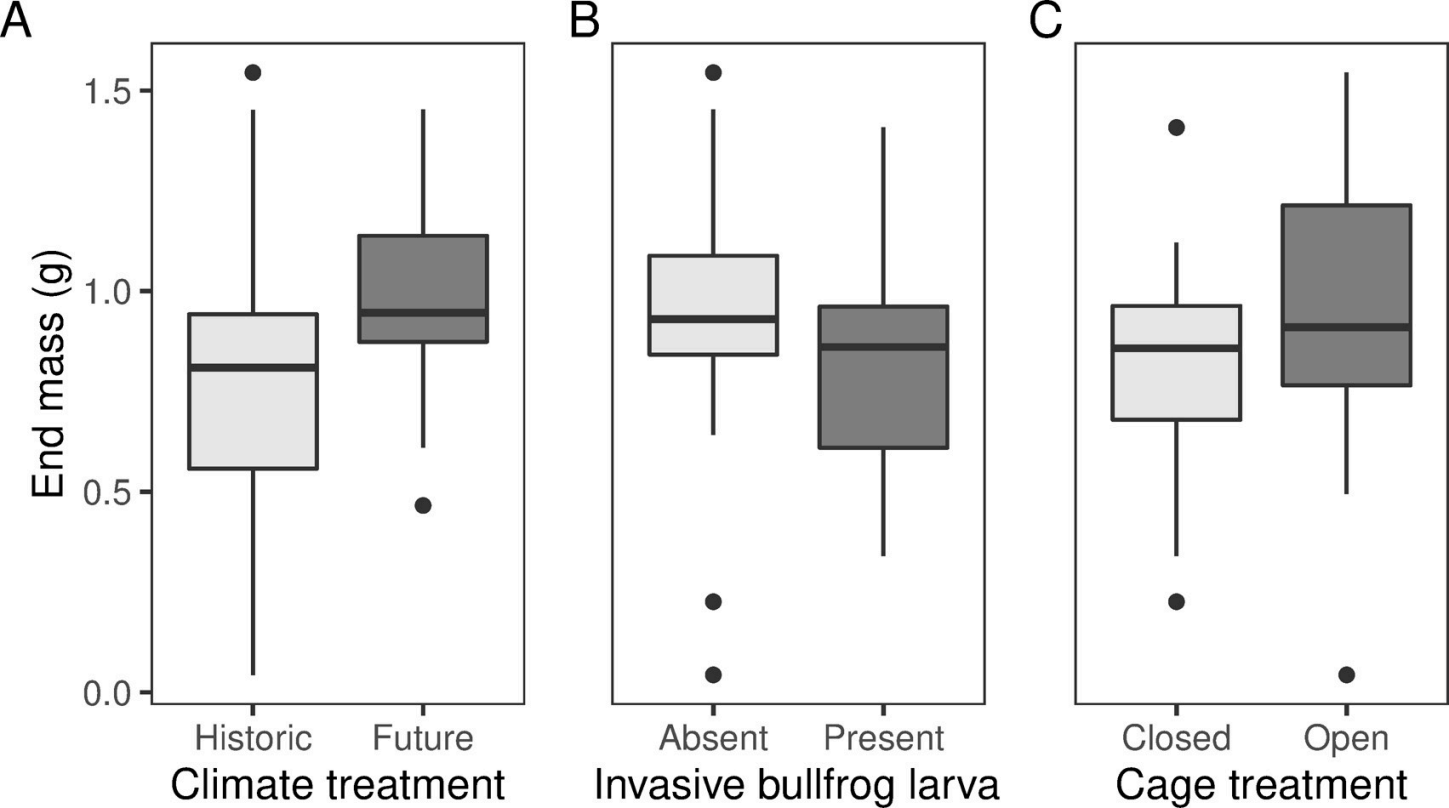
Pacific chorus frog weight at metamorphosis (gosner stage 42). A) Pacific chorus frog larvae were heavier at stage 42 [49] under simulated future climate conditions (p = 0.04). B) Pacific chorus frog larvae metamorphosed at a heavier weight in the absence of bullfrog larva (p = 0.03). C) Pacific chorus frog larvae were heavier in open cage treatments at stage 42 (p = 0.008). Boxplots are standard boxplots, with median and standard quartiles ± 1.5*IQR whiskers. Data were analyzed using a Gaussian GLM with a log link.

Pacific chorus frog mass at stage 42 was affected by climate, invasive species presence, cage treatment, and starting body length (Table 1B, full regression results in Table B in S2 File). Pacific chorus frogs were on average 0.23 g (29.2%) heavier at stage 42 under future climates (p = 0.04; Fig 4A). When invasive bullfrog tadpoles were present, Pacific chorus frogs weighed less at stage 42, an average of 0.13 g (16.4%) lighter (p = 0.03; Fig 4B). Similarly, Pacific chorus frogs weighed an average of 0.14 g (17.2%) less in closed cage treatments (p = 0.008, Fig 4C). Pacific chorus frog tadpoles that entered the experiment at a longer body length emerged heavier overall (p < 0.0001).

**Fig 5:**
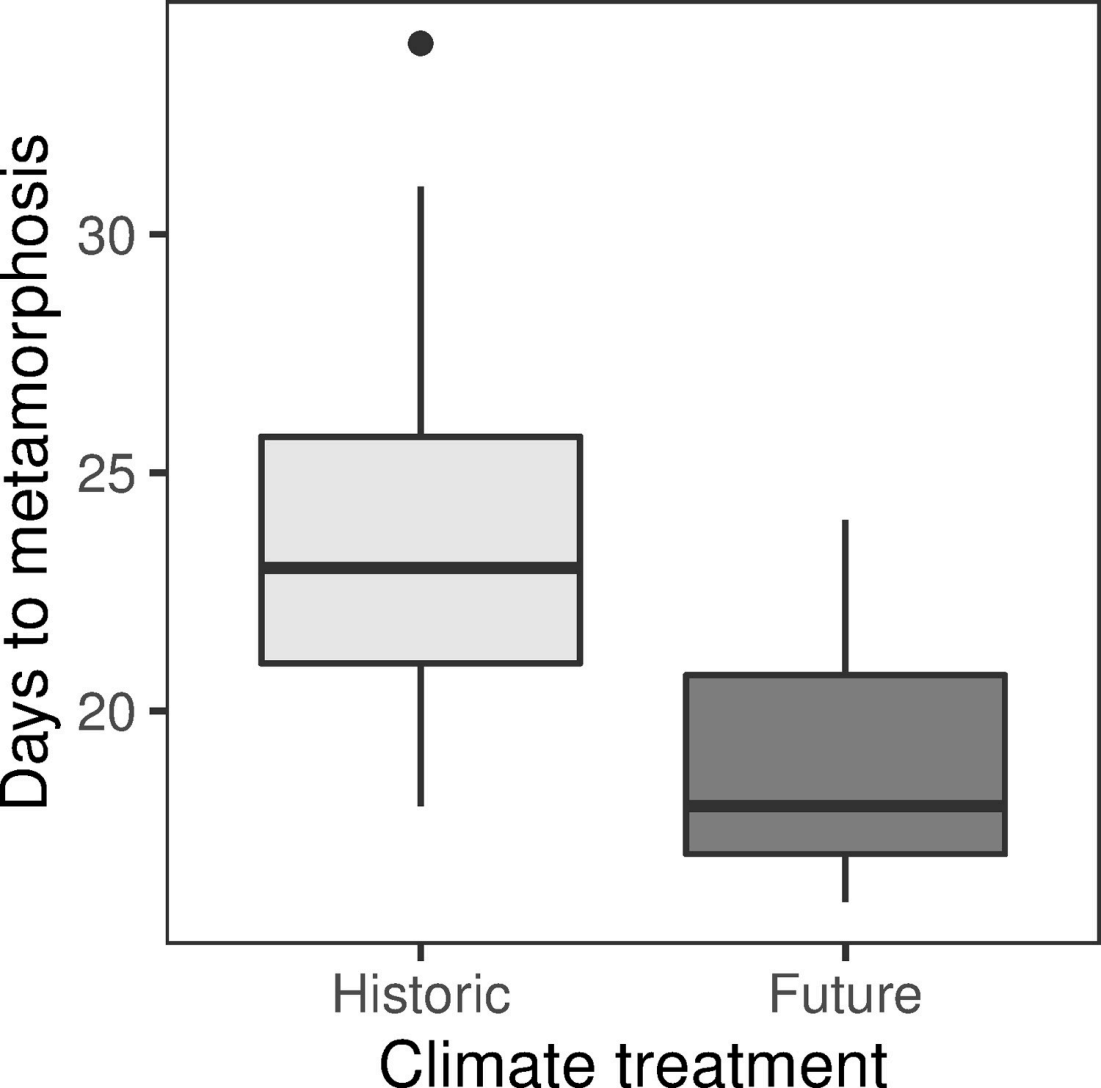
Pacific chorus frog development under simulated future conditions. Pacific chorus frog larvae reached metamorphosis (Gosner stage 42 [49]) an average of 5 days sooner under future climate conditions (quasi-Poisson GLM with log link; p<0.0001). Boxplots are standard boxplots, with median and standard quartiles ± 1.5*IQR whiskers.

Climate and beginning body length influenced days to stage 42 (metamorphosis; Table 1C, full regression results in Table C in S2 File). Pacific chorus frog tadpoles under future climate treatments reached stage 42 an average of 5 days sooner (p < 0.0001, Fig 5). Tadpoles that entered the experiment at a larger body size metamorphosed sooner (p < 0.0001).

## Discussion

Bullfrogs had the largest negative impact on our measured outcomes, with reductions in mass at stage 42 and relative growth through stage 42 when combined with future climate. Marginal evidence for a reduction in relative growth rate was also detected, suggesting that a larger sample size would be needed to detect the proportionally smaller daily differences due to experimental treatments. Although other studies have reported reduced survival of Pacific chorus frogs in the presence of larval bullfrogs (e.g., small tanks in [39, 43]), we did not see reduced survival due to bullfrog presence even under future climate conditions. Overwintered bullfrogs may counterintuitively have a smaller effect on competitors than first year bullfrog tadpoles [43, 60], suggesting that larger negative effects of bullfrog tadpoles may be seen if the experiment were repeated with first year tadpoles of a more similar size to the Pacific chorus frog tadpoles in this experiment. Kupferberg [37] suggests that bullfrog larvae have a negative effect on food availability, which could drive the difference in mass observed in that study. However, in our study, food was not limited, suggesting that direct competition for food did not explain the differences in mass when Pacific chorus frogs were paired with bullfrogs in our experiment. Furthermore, if competition for food drove the reduced mass, we would expect to see the Pacific chorus frogs in the closed treatments unaffected by bullfrog presence, which is not what we observed. It is possible that the presence of the bullfrog resulted in reduced activity of the chorus frogs [61], which could result in less foraging activity and lower mass gain. However, Kupferberg [37] did not see reduced foraging time in Pacific chorus frogs when in enclosures with bullfrogs. Similarly, Monello et al. [62] did not observe reduced activity in Pacific chorus frogs in the presence of bullfrogs. Instead, the chorus frogs raised with bullfrogs were more active when paired with a bullfrog, suggesting that Pacific chorus frogs in our study could have expended more energy via activity in tanks with bullfrogs. It is unclear, however, why the Pacific chorus frogs did not also consume more food to counter the additional energy expenditure. Although previous studies did not document changes in foraging behavior in response to bullfrog tadpoles [43, 62], studies have shown that Pacific chorus frog tadpoles modify foraging behavior based on resource availability and predator density [63, 64], suggesting some plasticity in foraging behavior based on environmental conditions and supporting the expectation of increased foraging based on energy needs. We did not conduct behavioral observations of the tadpoles in our study, so beyond the finding of reduced mass in the presence of bullfrog tadpoles, we do not have direct evidence of increased activity or changes in foraging behavior in Pacific chorus frog tadpoles in our study. It is possible that the presence of bullfrogs activated a stress response in the Pacific chorus frog tadpoles, resulting in reduced mass at metamorphosis.

The relative size and development of each species during the interaction and the presence of additional stressors can alter the effects of bullfrogs on native species like Pacific chorus frogs. Unlike Preston et al. [12], we did not see slower development of Pacific chorus frogs in tanks with bullfrogs. This difference may indicate that the effect of bullfrogs may depend on the developmental stage of the Pacific chorus frogs. Preston et al. [12] report a starting SVL of 7.43 mm, while our average starting SVL was 26.6 mm, suggesting that the tadpoles in our study were likely much further along in development. Other studies have found differing effects of environmental stressors based on the developmental stage of tadpoles [65]. Although our cage treatments were intended to test the differences between direct and indirect interactions (physical vs. chemical/visual) between chorus frogs and bullfrogs, evidence suggests that the physical space constraints posed by the closed cage treatment was itself a stressor, as tadpoles in closed treatments were lighter at stage 42 than tadpoles in open treatments. Tadpoles in closed cage treatments had less area to swim, which may have reduced foraging time, as has been observed in spadefoot toad tadpoles [66]. Similarly, relative growth was reduced under future climates only when bullfrogs were present. Taken together, bullfrogs have clear negative effects on native tadpole growth, both body length and body mass and this effect can be enhanced or driven by the interaction with additional stressors.

Future climate conditions affected native tadpole growth and development. Pacific chorus frogs developed faster in future climates, as expected, but surprisingly emerged at a heavier weight relative to the historic climate treatment (Fig A in S1 File). These results suggest that Pacific chorus frog tadpoles allocated energy to rapid development without a corresponding reduction in mass or body length, as has been observed in other studies [8, 67]. In a mesocosm study, warmer temperature and reduced hydroperiod did not negatively impact Pacific chorus frog size at metamorphosis, and the authors suggest the increase in periphyton growth due to warmer temperatures reduced the effects of competition [67]. It is likely that Pacific chorus frog growth and development were supported by the abundant food resources available in our experiment. Alternatively, it may be that our future climate conditions fell within the range of optimal thermal conditions for development (23-25°C) identified by Thurman and Garcia [8], resulting in heavier tadpoles under these warmer conditions. We explored this idea by summing the number of days above 23°C in future and historic treatment groups and found that, on average, tadpoles in the future climate treatments experienced 13.95 ±4.11 days above 23°C, compared to 1.6±2.2 days above 23°C in the historic treatment group. In addition, our climate treatments included nighttime cooling, which may have allowed tadpoles to avoid some negative effects of future climate scenarios observed in other studies which did not include diurnal temperature fluctuations (e.g., [8]). Small ponds can exhibit spikes in dissolved oxygen overnight due to differences between water temperature and air temperature and oxygen saturation in the water [68]. Because oxygen concentration in water depends on water temperature, allowing nighttime cooling may increase oxygen availability, reducing stress due to warmer daytime conditions. However, our results suggest that Pacific chorus frogs were able to respond plastically to future climate conditions, even in the presence of bullfrog larvae. Pacific chorus frog tadpoles in future climate conditions in the presence of a bullfrog larva had a lower relative growth, indicating a cost to developing in the presence of both stressors, but the difference in mass between the invasive species present group and the invasive species absent group was smaller compared to the historic treatment, suggesting the warmer temperatures in the future climate treatment may have reduced the impact of the invasive species on mass acquisition (Fig B in S1 File). We do not have thermal data for the pond where the chorus frogs were collected for this experiment, so our thermal regime may not represent precise historical conditions for this pond. It is also likely that future conditions may be more extreme than we have modeled them here, and in more extreme conditions we would likely see negative effects of future climate scenarios. Although the tadpoles in our study did not exhibit reduced growth rates under future climate conditions, energy allocation towards growth and development (progress towards metamorphosis) may result in reduced energy allocation toward immune function or other functions which were not observed in this experiment but could result in negative effects of future climate conditions in terrestrial life stages.

The effects of stressors in the larval environment can carry-over into the terrestrial environment. Size at metamorphosis can predict aspects of post-metamorphic biology in amphibians (but also see [69, 70]), including in Pacific chorus frogs [71]. For example, body length (SVL) at metamorphosis is correlated positively with maximum adult body size in many species [72]. Size at metamorphosis also impacts survival [73], physical performance [71, 74–76], metabolic rate [76], and fitness [77]. In our study, bullfrogs reduced the size of Pacific chorus frogs at stage 42, both in mass and body length (combined with other stressors), suggesting that the presence of this invasive species in the larval habitat may negatively affect terrestrial stages if compensatory growth does not occur after metamorphosis. Conversely, Pacific chorus frogs demonstrated potentially beneficial plasticity in response to future climate conditions in the absence of bullfrogs. Moreover, the maintenance of overall body condition (smaller but proportionally heavier metamorphs) when future climate conditions overlap with bullfrog presence suggests that Pacific chorus frogs may be partially compensating for the negative effects of bullfrogs via increased allocation of energy towards mass. Body length, however, can be an important indicator of performance, including jumping distance [78]. Thus, more research is needed to understand the potential tradeoffs in mass and body length in response to invasive bullfrogs under warmer temperatures. Strong plasticity, as demonstrated by Pacific chorus frog larvae in our study, may allow species to match the demands of new environments [79], including future climate change.

## Supporting information

S1 Appendix Description of climate scenarios. (PDF)

S1 Data Dataset upon which all analyses are based. (XLSX)

S1 File Supplemental figures A and B. (PDF)

S2 File Regression results from GLM analyses. (PDF)

## References

[1] Carpenter SR, Stanley EH, Vander Zanden MJ. State of the world’s freshwater ecosystems: physical, chemical, and biological changes. Annu Rev Environ Resour. 2011;36: 75–99. doi: 10.1146/annurev-environ-021810-094524

[2] Ali S, Mishra PK, Islam A, Alam NM. Simulation of water temperature in a small pond using parametric statistical models: implications of climate warming. J Environ Eng. 2016;142: 04015085. doi: 10.1061/(ASCE)EE.1943-7870.0001050

[3] Dybdahl MF, Kane SL. Adaptation vs. phenotypic plasticity in the success of a clonal invader. Ecology. 2005;86: 1592–1601.

[4] Lenz M, da Gama BAP, Gerner NV, Gobin J, Gröner F, Harry A, et al. Non-native marine invertebrates are more tolerant towards environmental stress than taxonomically related native species: Results from a globally replicated study. Environ Res. 2011;111: 943–952. doi: 10.1016/j.envres.2011.05.001 21632049

[5] Zerebecki RA, Sorte CJB. Temperature tolerance and stress proteins as mechanisms of invasive species success. PLOS ONE. 2011;6: e14806. doi: 10.1371/journal.pone.0014806 21541309 PMC3082523

[6] Blaustein AR, Walls SC, Bancroft BA, Lawler JJ, Searle CL, Gervasi SS. Direct and indirect effects of climate change on amphibian populations. Diversity. 2010;2: 281–313. doi: 10.3390/d2020281

[7] Ficetola GF, Maiorano L. Contrasting effects of temperature and precipitation change on amphibian phenology, abundance and performance. Oecologia. 2016;181: 683–693. doi: 10.1007/s00442-016-3610-9 27008454

[8] Thurman LL, Garcia TS. Differential plasticity in response to simulated climate warming in a high-elevation amphibian assemblage. J Herpetol. 2017;51: 232–239. doi: 10.1670/16-502

[9] Cayuela H, Arsovski D, Thirion J-M, Bonnaire E, Pichenot J, Boitaud S, et al. Demographic responses to weather fluctuations are context dependent in a long-lived amphibian. Glob Change Biol. 2016;22: 2676–2687. doi: 10.1111/gcb.13290 27002592

[10] Ficetola GF, Siesa ME, Manenti R, Bottoni L, Bernardi FD, Padoa‐Schioppa E. Early assessment of the impact of alien species: differential consequences of an invasive crayfish on adult and larval amphibians. Divers Distrib. 2011;17: 1141–1151. doi: 10.1111/j.1472-4642.2011.00797.x

[11] Lillo F, Faraone FP, Lo Valvo M. Can the introduction of *Xenopus laevis* affect native amphibian populations? Reduction of reproductive occurrence in presence of the invasive species. Biol Invasions. 2011;13: 1533–1541. doi: 10.1007/s10530-010-9911-8

[12] Preston DL, Henderson JS, Johnson PTJ. Community ecology of invasions: direct and indirect effects of multiple invasive species on aquatic communities. Ecology. 2012;93: 1254–1261. doi: 10.1890/11-1821.1 22834365

[13] Bucciarelli GM, Blaustein AR, Garcia TS, Kats LB. Invasion complexities: the diverse impacts of nonnative species on amphibians. Copeia. 2014;2014: 611–632.

[14] Nunes AL, Fill JM, Davies SJ, Louw M, Rebelo AD, Thorp CJ, et al. A global meta-analysis of the ecological impacts of alien species on native amphibians. Proc R Soc B Biol Sci. 2019;286: 20182528. doi: 10.1098/rspb.2018.2528 30963838 PMC6408899

[15] Falaschi M, Melotto A, Manenti R, Ficetola GF. Invasive species and amphibian conservation. Herpetologica. 2020;76: 216–227. doi: 10.1655/0018-0831-76.2.216

[16] Denver RJ, Middlemis-Maher J. Lessons from evolution: developmental plasticity in vertebrates with complex life cycles. J Dev Orig Health Dis. 2010;1: 282–291. doi: 10.1017/S2040174410000279 25141931

[17] Marino JA Jr., Werner EE. Synergistic effects of predators and trematode parasites on larval green frog (*Rana clamitans*) survival. Ecology. 2013;94: 2697–2708. doi: 10.1890/13-0396.1 24597217

[18] Florencio M, Burraco P, Rendón MÁ, Díaz-Paniagua C, Gomez-Mestre I. Opposite and synergistic physiological responses to water acidity and predator cues in spadefoot toad tadpoles. Comp Biochem Physiol A Mol Integr Physiol. 2020;242: 110654. doi: 10.1016/j.cbpa.2020.110654 31926298

[19] Relyea RA. Synergistic impacts of malathion and predatory stress on six species of North American tadpoles. Environ Toxicol Chem. 2004;23: 1080–1084. doi: 10.1897/03-259 15095908

[20] Bancroft BA, Baker NJ, Blaustein AR. A meta-analysis of the effects of ultraviolet B radiation and its synergistic interactions with pH, contaminants, and disease on amphibian survival. Conserv Biol. 2008;22: 987–996. doi: 10.1111/j.1523-1739.2008.00966.x 18616747

[21] Relyea RA. Morphological and behavioral plasticity of larval anurans in response to different predators. Ecology. 2001;82: 523–540. doi: 10.2307/2679877

[22] Relyea RA. Fine-tuned phenotypes: tadpole plasticity under 16 combinations of predators and competitors. Ecology. 2004;85: 172–179.

[23] Garcia TS, Stacy J, Sih A. Larval salamander response to UV radiation and predation risk: color change and microhabitat use. Ecol Appl. 2004;14: 1055–1064. doi: 10.1890/02-5288

[24] Van Buskirk J, McCollum SA. Influence of tail shape on tadpole swimming performance. J Exp Biol. 2000;203: 2149–2158. doi: 10.1242/jeb.203.14.2149 10862727

[25] Relyea RA. Getting out alive: how predators affect the decision to metamorphose. Oecologia. 2007;152: 389–400. doi: 10.1007/s00442-007-0675-5 17356812

[26] Tejedo M, Reques R. Plasticity in metamorphic traits of natterjack tadpoles: the interactive effects of density and pond duration. Oikos. 1994;71: 295–304. doi: 10.2307/3546278

[27] Merilä J, Laurila A, Pahkala M, Räsänen K, Laugen AT. Adaptive phenotypic plasticity in timing of meta- morphosis in the common frog *Rana temporaria*. Écoscience 2000;7: 18–24.

[28] Álvarez D, Nicieza AG. Effects of temperature and food quality on anuran larval growth and metamorphosis. Funct Ecol. 2002;16: 640–648. doi: 10.1046/j.1365-2435.2002.00658.x

[29] Semlitsch RD. Relationship of pond drying to the reproductive success of the salamander *Ambystoma talpoideum*. Copeia. 1987;1987: 61–69. doi: 10.2307/1446038

[30] Benard MF. Survival trade-offs between two predator-induced phenotypes in Pacific treefrogs (*Pseudacris regilla*). Ecology. 2006;87: 340–346. doi: 10.1890/05-0381 16637360

[31] Koprivnikar J, Paull SH, Johnson PTJ. Combined influence of hydroperiod and parasitism on larval amphibian development. Freshw Sci. 2014;33: 941–949. doi: 10.1086/676674

[32] Li Y, Ke Z, Wang Y, Blackburn TM. Frog community responses to recent American bullfrog invasions. Curr Zool. 2011;57: 83–92. doi: 10.1093/czoolo/57.1.83

[33] Lannoo M, editor. Amphibian Declines: The Conservation Status of United States Species. 1st ed. University of California Press; 2005. Available: http://www.jstor.org/stable/10.1525/j.ctt1pp5xd

[34] Kats LB, Ferrer RP. Alien predators and amphibian declines: review of two decades of science and the transition to conservation. Divers Distrib. 2003;9: 99–110.

[35] Jancowski K, Orchard S. Stomach contents from invasive American bullfrogs *Rana catesbeiana* (= *Lithobates catesbeianus*) on southern Vancouver Island, British Columbia, Canada. NeoBiota. 2013;16: 17–37. doi: 10.3897/neobiota.16.3806

[36] Kiesecker JM, Blaustein AR. Effects of introduced bullfrogs and smallmouth bass on microhabitat use, growth, and survival of native red-legged frogs (*Rana aurora*). Conserv Biol. 1998;12: 776–787.

[37] Lawler SP, Dritz D, Strange T, Holyoak M. Effects of introduced mosquitofish and bullfrogs on the threatened California red-legged frog. Conserv Biol. 1999;13: 613–622. doi: 10.1046/j.1523-1739.1999.98075.x

[38] Boone MD, Little EE, Semlitsch RD. Overwintered bullfrog tadpoles negatively affect salamanders and anurans in native amphibian communities. Copeia. 2004;2004: 683–690. doi: 10.1643/CE-03-229R1

[39] Blaustein AR, Jones DK, Urbina J, Cothran RD, Harjoe C, Mattes B, et al. Effects of invasive larval bullfrogs (*Rana catesbeiana*) on disease transmission, growth and survival in the larvae of native amphibians. Biol Invasions. 2020 [cited 28 Feb 2020]. doi: 10.1007/s10530-020-02218-4

[40] Adams MJ. Pond permanence and the effects of exotic vertebrates on anurans. Ecol Appl. 2000;10: 559–568. doi: 10.1890/1051-0761(2000)010[0559:PPATEO]2.0.CO;2

[41] Govindarajulu PP, Anholt BR. Interaction between biotic and abiotic factors determines tadpole survival rate under natural conditions. Écoscience. 2006;13: 413–421. doi: 10.2980/i1195-6860-13-3-413.1

[42] Govindarajulu P. Introduced bullfrogs (*Rana catesbeiana*) in British Columbia: Impacts on native Pacific treefrogs (*Hyla regilla*) and red-legged frogs (*Rana aurora*). University of Victoria. 2004. doi: 10.1530/rep.1.00067

[43] Kupferberg SJ. Bullfrog (*Rana catesbeiana*) invasion of a California river: the role of larval competition. Ecology. 1997;78: 1736–1751. doi: 10.2307/2266097

[44] Bury RB, Whelan JA. Ecology and management of the bullfrog. Washington, D.C.; 1985. Report No.: 155. Available: http://pubs.er.usgs.gov/publication/rp155

[45] Lucid MK, Robinson L, Ehlers SE. Chapter 3. Amphibians—Multispecies Baseline Initiative. Coeur d’Alene, ID, USA: Idaho Department of Fish and Game; 2016 pp. 104–147.

[46] Tylianakis JM, Didham RK, Bascompte J, Wardle DA. Global change and species interactions in terrestrial ecosystems. Ecol Lett. 2008;11: 1351–1363. doi: 10.1111/j.1461-0248.2008.01250.x 19062363

[47] Griffith S, Bancroft BA. Bancroft-Lab-Temperature Control System Repository. 2021. Available: https://github.com/scott-whitworth/bancroft_temperature_control

[48] Lee S-Y, Ryan ME, Hamlet AF, Palen WJ, Lawler JJ, Halabisky M. Projecting the hydrologic impacts of climate change on montane wetlands. PLOS ONE. 2015;10: e0136385. doi: 10.1371/journal.pone.0136385 26331850 PMC4557981

[49] Gosner KL. A simplified table for staging anuran embryos and larvae with notes on identification. Herpetologica. 1960;16: 183–190.

[50] Schneider CA, Rasband WS, Eliceiri KW. NIH Image to ImageJ: 25 years of image analysis. Nat Methods. 2012;9: 671–675. doi: 10.1038/nmeth.2089 22930834 PMC5554542

[51] Annibale FS, de Sousa VTT, de Sousa CE, Venesky MD, Rossa-Feres D de C, Nomura F, et al. Influence of substrate orientation on tadpoles feeding efficiency. Biol Open. 2018; bio.037598. doi: 10.1242/bio.037598 30578249 PMC6361219

[52] R Core Team. R: A language and environment for statistical computing. Vienna, Austria: R Foundation for Statistical Computing; 2020. Available: https://www.R-project.org/

[53] Fox J, Weisberg S, Price B, Adler D, Bates D, Baud-Bovy G, et al. car: companion to applied regression. 2021. Available: https://CRAN.R-project.org/package=car

[54] Agresti A. An Introduction to Categorical Data Analysis. Wiley; 2007.

[55] Lenth RV, Buerkner P, Herve M, Love J, Riebl H, Singmann H. emmeans: estimated marginal means, aka least-squares means. 2021. Available: https://CRAN.R-project.org/package=emmeans

[56] Wickham H, Chang W, Henry L, Pedersen TL, Takahashi K, Wilke C, et al. ggplot2: create elegant data visualisations using the grammar of graphics. 2020. Available: https://CRAN.R-project.org/package=ggplot2

[57] Pedersen TL. patchwork: the composer of plots. 2020. Available: https://CRAN.R-project.org/package=patchwork

[58] Walker DA, Smith TJ. Logistic regression under sparse data conditions. J Mod Appl Stat Methods. 2019;18: 2–18. doi: 10.22237/jmasm/1604190660

[59] Kosmidis I, Pagui ECK, Konis K, Sartori N. brglm2: bias reduction in generalized linear models. 2021. Available: https://CRAN.R-project.org/package=brglm2

[60] Werner EE. Ontogenetic scaling of competitive relations: size-dependent effects and responses in two anuran larvae. Ecology. 1994;75: 197–213. doi: 10.2307/1939394

[61] Melotto A, Ficetola GF, Alari E, Romagnoli S, Manenti R. Visual recognition and coevolutionary history drive responses of amphibians to an invasive predator. Behav Ecol. 2021;32: 1352–1362. doi: 10.1093/beheco/arab101

[62] Monello RJ, Dennehy JJ, Murray DL, Wirsing AJ. Growth and behavioral responses of tadpoles of two native frogs to an exotic competitor, *Rana catesbeiana*. J Herpetol. 2006;40: 403–407.

[63] Hammond JI, Luttbeg B, Sih A. Predator and prey space use: dragonflies and tadpoles in an interactive game. Ecology. 2007;88: 1525–1535. doi: 10.1890/06-1236 17601144

[64] Luttbeg B, Hammond JI, Sih A. Dragonfly larvae and tadpole frog space use games in varied light conditions. Behav Ecol. 2009;20: 13–21. doi: 10.1093/beheco/arn107

[65] Crespi EJ, Denver RJ. Roles of stress hormones in food intake regulation in anuran amphibians throughout the life cycle. Comp Biochem Physiol A Mol Integr Physiol. 2005;141: 381–390. doi: 10.1016/j.cbpb.2004.12.007 16140236

[66] Denver RJ, Mirhadi N, Phillips M. Adaptive plasticity in amphibian metamorphosis: response of *Scaphiopus hammondii* tadpoles to habitat desiccation. Ecology. 1998;79: 1859–1872. doi: 10.1890/0012-9658(1998)079[1859:APIAMR]2.0.CO;2

[67] O’Regan SM, Palen WJ, Anderson SC. Climate warming mediates negative impacts of rapid pond drying for three amphibian species. Ecology. 2014;95: 845–855. doi: 10.1890/13-0916.1 24933805

[68] Holgerson MA, Zappa CJ, Raymond PA. Substantial overnight reaeration by convective cooling discovered in pond ecosystems. Geophys Res Lett. 2016;43: 8044–8051. doi: 10.1002/2016GL070206

[69] Schmidt BR, Hödl W, Schaub M. From metamorphosis to maturity in complex life cycles: equal performance of different juvenile life history pathways. Ecology. 2012;93: 657–667. doi: 10.1890/11-0892.1 22624219

[70] Earl JE, Whiteman HH. Are commonly used fitness predictors accurate? A meta-analysis of amphibian size and age at metamorphosis. Copeia. 2015;103: 297–309. doi: 10.1643/CH-14-128

[71] Watkins TB. A quantitative genetic test of adaptive decoupling across metamorphosis for locomotor and life-history traits in the Pacific tree frog, *Hyla regilla*. Evolution. 2001;55: 1668–1677. doi: 10.1111/j.0014-3820.2001.tb00686.x 11580026

[72] Werner EE. Amphibian metamorphosis: growth rate, predation risk, and the optimal size at transformation. Am Nat. 1986;128: 319–341.

[73] Székely D, Cogălniceanu D, Székely P, Armijos-Ojeda D, Espinosa-Mogrovejo V, Denoël M. How to recover from a bad start: size at metamorphosis affects growth and survival in a tropical amphibian. BMC Ecol. 2020;20: 24. doi: 10.1186/s12898-020-00291-w 32316956 PMC7175581

[74] John-Alder HB, Morin PJ. Effects of larval density on jumping ability and stamina in newly metamorphosed *Bufo woodhousii fowleri*. Copeia. 1990;1990: 856–860. doi: 10.2307/1446453

[75] Goater CP, Semlitsch RD, Bernasconi MV. Effects of body size and parasite infection on the locomotory performance of juvenile toads, *Bufo bufo*. Oikos. 1993;66: 129–136. doi: 10.2307/3545205

[76] Beck CW, Congdon JD. Effects of age and size at metamorphosis on performance and metabolic rates of Southern Toad, *Bufo terrestris*, metamorphs. Funct Ecol. 2000;14: 32–38. doi: 10.1046/j.1365-2435.2000.00386.x

[77] Semlitsch RD, Scott DE, Pechmann JHK. Time and size at metamorphosis related to adult fitness in *Ambystoma talpoideum*. Ecology. 1988;69: 184–192. doi: 10.2307/1943173

[78] Orizaola G, Laurila A. Microgeographic variation in the effects of larval temperature environment on juvenile morphology and locomotion in the pool frog. J Zool. 2009;277: 267–274. doi: 10.1111/j.1469-7998.2008.00530.x

[79] Urban MC, Richardson JL, Freidenfelds NA. Plasticity and genetic adaptation mediate amphibian and reptile responses to climate change. Evol Appl. 2013;7: 88–103. doi: 10.1111/eva.12114 24454550 PMC3894900

