# Supplementary material for: Effects of elevated temperature, reduced hydroperiod, and invasive bullfrog larvae on pacific chorus frog larvae
Source: PLoS One. 2022 Mar 15;17(3):e0265345. doi: 10.1371/journal.pone.0265345 (PMC8923472; doi:10.1371/journal.pone.0265345)
Supplement: S1 Appendix — (PDF) [file pone.0265345.s001.pdf]

Temperature scenarios

Future projections for water temperature are not available for this region, so we used the relationship between air temperature and water temperature measured at three nearby ponds where Pacific chorus frogs have been observed to convert daily maximum and minimum air temperatures into daily maximum and minimum water temperatures (Figure S1, below).

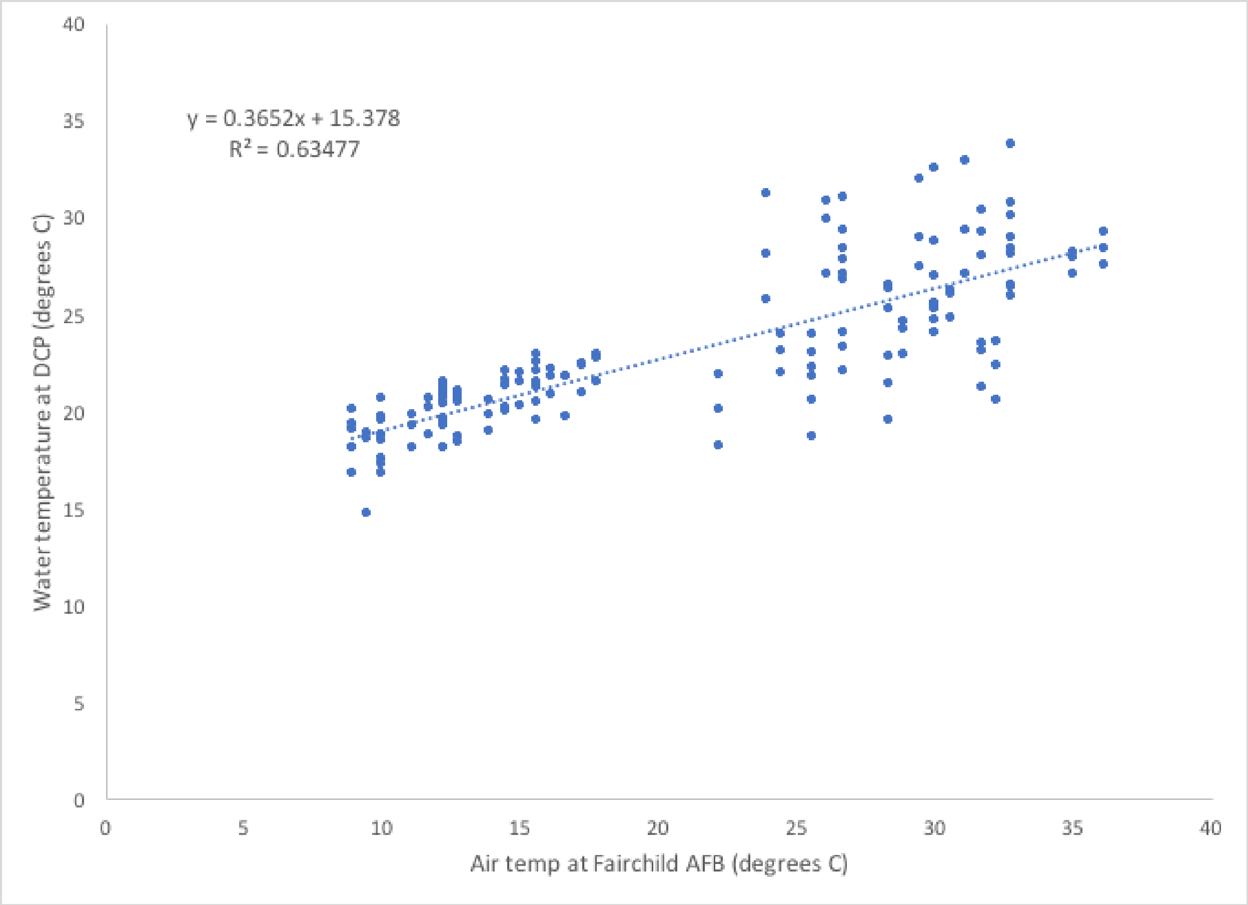

Figure S1. Relationship between water temperature and air temperature in three ponds near Reardan, WA. In three ponds, two Hobo pendant data loggers were deployed from June 21, 2017-July 18, 2017. Data loggers were deployed 6 cm below the water surface and 50 cm below water surface to encompass the range of temperatures observed in the ponds. Water temperature data are daily maximum and daily minimum for each data logger from each pond. Air

temperature is the recorded daily maximum and minimum temperature at a nearby location (Fairchild Air Force Base, USA).

To obtain comparable future and historic air temperature data for our region, we downloaded historic (1976-1999) and future (2070-2090) daily maximum and minimum temperature data from the Downscaled CMIP3 and CHIP5 Climate and Hydrology Projections archive at http://gdo-dcp.ucllnl.org/ for the Spokane River tributary area for the months May-September. We downloaded the BCCAv2 CMIP5 data from all available climate models. To generate daily historic maximum and minimum temperatures for each day of our experiment, we randomly selected one maximum temperature and one minimum temperature for each calendar day (e.g., June 30th) from the historic data. These max and mins were independent of each other (e.g., the max may be from 1979, while the min is from 1986, but both on June 30th). We used projected future data for 2070-2090 under RCP 8.5 to generate daily future (warmer) daily maximum and minimum temperatures. We calculated a simple mean ensemble of all model projections for a given day in a given future year for both Tmax and Tmin. We then used R [1] to randomly select one future max and one future min for each calendar day in our experiment. Because the future data were from an ensembled model, the variation in daily max temperatures was lower relative to the randomly selected (non-ensemble) historic data. We therefore calculated the average difference between the future and historic data for both daily maximum and daily minimum and then subtracted that value from the future projections so the two temperature treatments would have the same scale of day-to-day variation. The mean difference between the future daily maximum and the historic daily maximum was 2.24^{o}C and the mean difference between the minima was 1.45^{o}C.

Hydroperiod scenarios

Water withdrawal amounts were determined using published data for montane environments [2] combined with historic and projected open water PET data http://gdo-dcp.ucllnl.org/. Data were extracted from Lee et al. (2015) to determine historic evaporation rates (1.2 cm/week) and then the relative historic and future rates were calculated from the PET data, specifically by using data from 1979-1999 in the months of May, June, July, and August and data from 2070-2090 under RCP 8.5 and calculating the percent difference between PET for these two time periods. Our experiment was four weeks long and we used rates in June and July to reflect the timing of our experiment. Extraction volumes are shown in Table 1.

Table 1. Water extraction values for simulating hydroperiod. Each tank started with water 15 cm deep. Water was removed using a small beaker on the same day each week.

Treatment	Week	Starting water level	Amount removed	Ending water level
Future	1	15	2.2	12.8
	2	12.8	2.1	10.8
	3	10.8	1.9	8.9
	4	8.9	1.8	7.1
Historic	1	15	1.2	13.8
	2	13.8	1.2	12.6
	3	12.6	1.2	11.4
	4	11.4	1.2	10.2

1. R Core Team. R: A language and environment for statistical computing. Vienna, Austria: R Foundation for Statistical Computing; 2020. Available: https://www.R-project.org/

2. Lee S-Y, Ryan ME, Hamlet AF, Palen WJ, Lawler JJ, Halabisky M. Projecting the Hydrologic Impacts of Climate Change on Montane Wetlands. PLOS ONE. 2015;10: e0136385. doi:10.1371/journal.pone.0136385
