# Supplementary material for: Effects of elevated temperature, reduced hydroperiod, and invasive bullfrog larvae on pacific chorus frog larvae
Source: PLoS One. 2022 Mar 15;17(3):e0265345. doi: 10.1371/journal.pone.0265345 (PMC8923472; doi:10.1371/journal.pone.0265345)
Supplement: S1 File — (PDF) [file pone.0265345.s003.pdf]

Tasker et al. Supplemental Figures

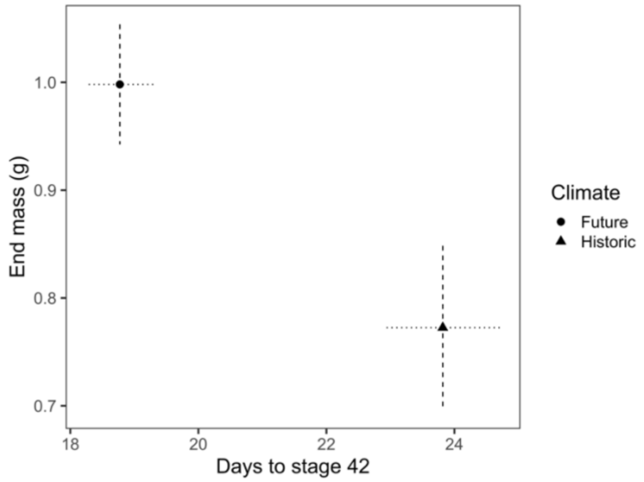

Figure A. Comparison of patterns in development and end mass (g) in Pacific chorus frog tadpoles under two climate regimes. Tadpoles under warmer, more rapidly drying conditions (Future treatment) developed faster (fewer days to stage 42) and emerged heavier compared to tadpoles in the Historic climate treatment. Data are group means + 1 SE for both days to stage 42 (dotted horizontal error bars) and end weight (dashed vertical error bars).

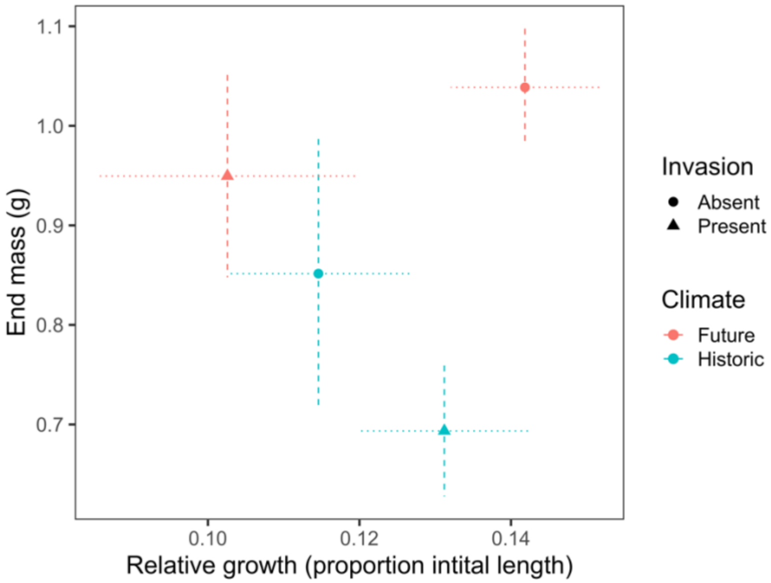

Figure B. Comparison of patterns in absolute relative growth (proportion change in initial length) and end mass (g) in Pacific chorus frog tadpoles under two climate levels and two invasion levels. Under future climate conditions, the presence of a bullfrog tadpole reduced relative growth more than mass, resulting in shorter, but heavier tadpoles. Data are group

means $\pm$ 1 SE for both end length (dotted horizontal error bars) and end weight (dashed vertical error bars).
