# Supplementary material for: Effects of elevated temperature, reduced hydroperiod, and invasive bullfrog larvae on pacific chorus frog larvae
Source: PLoS One. 2022 Mar 15;17(3):e0265345. doi: 10.1371/journal.pone.0265345 (PMC8923472; doi:10.1371/journal.pone.0265345)
Supplement: S2 File — (PDF) [file pone.0265345.s004.pdf]

Tasker et al. Supplemental Tables

Table A. Results of logistic regression model of treatment effects on survival.

Main effect	Coefficient	SE of coefficient	Z	p
Intercept	0.1327	5.4045	0.025	0.980
Climate (future)	-0.3514	0.8651	-0.406	0.685
Invasive (present)	-1.4930	0.9458	-1.215	0.224
Cage (open)	0.3474	0.8698	0.399	0.690
Start size	-0.5756	2.0385	-0.282	0.778

Table B. Regression results from gaussian GLM with log link model for mass (g) of Pacific chorus frogs at stage 42

Effect parameter	Coefficient	SE of coefficient	t-value	p-value
Intercept	-2.52443	0.73410	-3.439	0.00153
Climate (future)	0.14103	0.21500	0.656	0.51615
Invasive (present)	-0.01817	0.25459	-0.071	0.94350
Cage (open)	0.26961	0.20878	1.291	0.20504
Start size	0.83741	0.26703	3.136	0.00346
Climate (future): invasive (present)	-0.14239	0.32039	-0.444	0.65946
Climate (future): cage (open)	0.11785	0.27330	0.431	0.66896
Invasive (present): cage (open)	-0.26439	0.32334	-0.818	0.41906
Climate (future): invasive (present): cage (open)	0.14432	0.40990	0.352	0.72689

Table C. Regression results from quasi-Poisson GLM with log link model for development (days to stage 42) of Pacific chorus frogs

Effect parameter	Coefficient	SE of coefficient	t-value	p-value
Intercept	4.40303	0.29133	15.113	< 0.00001
Climate (future)	-0.27304	0.08359	-3.267	0.002440
Invasive (present)	-0.05409	0.07906	-0.684	0.498351
Cage (open)	-0.09466	0.07736	-1.224	0.229268
Start size	-0.45042	0.11079	-4.066	0.000258
Climate (future): invasive (present)	0.09901	0.11928	0.830	0.412120
Climate (future): cage (open)	0.07109	0.11900	0.597	0.554115
Invasive (present): cage (open)	0.06557	0.10912	0.601	0.551771
Climate (future): invasive (present): cage (open)	-0.03748	0.16637	-0.225	0.823076
